# Integrated molecular characterisation of endometrioid ovarian carcinoma identifies opportunities for stratification

**DOI:** 10.1038/s41698-021-00187-y

**Published:** 2021-06-02

**Authors:** Robert L. Hollis, Barbara Stanley, John P. Thomson, Michael Churchman, Ian Croy, Tzyvia Rye, Clare Bartos, Fiona Nussey, Melanie Mackean, Alison M. Meynert, Colin A. Semple, Charlie Gourley, C. Simon Herrington

**Affiliations:** 1 Nicola Murray Centre for Ovarian Cancer Research, Cancer Research UK Edinburgh Centre, MRC Institute of Genetics and Cancer, University of Edinburgh, Edinburgh, UK; 2 Beatson West of Scotland Cancer Centre and University of Glasgow, Glasgow, UK; 3 Edinburgh Cancer Centre, Western General Hospital, Edinburgh, UK; 4 MRC Human Genetics Unit, MRC Institute of Genetics and Cancer, University of Edinburgh, Edinburgh, UK

**Keywords:** Ovarian cancer, Ovarian cancer, Tumour biomarkers, Prognostic markers

## Abstract

Endometrioid ovarian carcinoma (EnOC) is an under-investigated ovarian cancer type. Recent studies have described disease subtypes defined by genomics and hormone receptor expression patterns; here, we determine the relationship between these subtyping layers to define the molecular landscape of EnOC with high granularity and identify therapeutic vulnerabilities in high-risk cases. Whole exome sequencing data were integrated with progesterone and oestrogen receptor (PR and ER) expression-defined subtypes in 90 EnOC cases following robust pathological assessment, revealing dominant clinical and molecular features in the resulting integrated subtypes. We demonstrate significant correlation between subtyping approaches: PR-high (PR + /ER + , PR + /ER−) cases were predominantly *CTNNB1*-mutant (73.2% vs 18.4%, *P* < 0.001), while PR-low (PR−/ER + , PR−/ER−) cases displayed higher *TP53* mutation frequency (38.8% vs 7.3%, *P* = 0.001), greater genomic complexity (*P* = 0.007) and more frequent copy number alterations (*P* = 0.001). PR-high EnOC patients experience favourable disease-specific survival independent of clinicopathological and genomic features (HR = 0.16, 95% CI 0.04–0.71). *TP53* mutation further delineates the outcome of patients with PR-low tumours (HR = 2.56, 95% CI 1.14–5.75). A simple, routinely applicable, classification algorithm utilising immunohistochemistry for PR and p53 recapitulated these subtypes and their survival profiles. The genomic profile of high-risk EnOC subtypes suggests that inhibitors of the MAPK and PI3K-AKT pathways, alongside PARP inhibitors, represent promising candidate agents for improving patient survival. Patients with PR-low *TP53*-mutant EnOC have the greatest unmet clinical need, while PR-high tumours—which are typically *CTNNB1*-mutant and *TP53* wild-type—experience excellent survival and may represent candidates for trials investigating de-escalation of adjuvant chemotherapy to agents such as endocrine therapy.

## Introduction

Ovarian cancer remains one of the most lethal malignancies in the developed world^1^. Endometrioid ovarian carcinoma (EnOC) is a unique ovarian cancer type, with distinct clinical and molecular characteristics^2,3^, accounting for around 10% of ovarian carcinoma diagnoses. A large number of EnOC patients are diagnosed at early stage and display excellent survival following maximal surgical cytoreduction^4,5^; however, a minority present with aggressive advanced-stage disease and suffer poor clinical outcome.

Currently, decisions regarding post-operative systemic chemotherapy for EnOC are made within a similar framework to other ovarian carcinoma histotypes: platinum-based chemotherapy, often in combination with paclitaxel, is offered to most patients with advanced stage disease and a minority of early stage cases, depending on tumour grade and sub-stage^6^. Identification of EnOC subtypes with distinct clinical behaviour has the potential to highlight cases for which new treatment options are needed to improve survival; molecular characterisation represents an opportunity to identify novel therapeutic vulnerabilities of high-risk and advanced-stage cases to biologically targeted agents, while also highlighting low-risk cases for which de-escalation of chemotherapy may be considered.

Early molecular studies characterising the biology of EnOC have been confounded by the inclusion of misclassified tumours now known to represent variants of high grade serous ovarian carcinoma (HGSOC), which is associated with markedly poorer overall prognosis and a distinct molecular landscape compared to EnOC^4,7–9^. While HGSOC can bear morphological resemblance to high grade EnOC, immunohistochemistry (IHC) for Wilms’ tumour 1 (WT1) has emerged as a useful discriminator between true EnOC (WT1-negative) and pseudo-endometrioid HGSOC (WT1-positive)^10^, and several studies have demonstrated its utility for improving the fidelity of EnOC diagnosis^11–14^.

In contrast to HGSOC, genomic studies of relatively small EnOC cohorts have identified a relatively low *TP53* mutation (*TP53*m) rate and a high frequency of *ARID1A*, *CTNNB1*, *PTEN*, *PIK3CA* and *KRAS* mutation^15–17^. Early mixed-histology transcriptomic studies of ovarian carcinoma reported that a proportion of EnOC cluster alongside HGSOC^18^; while these reports likely contained occult pseudo-endometrioid HGSOC masquerading as true EnOC, a recent report of 36 cases identified a proportion of contemporarily-defined EnOC that bear the high *TP53*m and extensive copy number alteration (CNA) burden reminiscent of HGSOC^19^. Though this study did not use routine WT1 IHC to exclude potential HGSOC cases, these data suggest the existence of a true EnOC subgroup with genomic features redolent of HGSOC. Moreover, recent IHC-based studies have identified aberrant p53 expression patterns, indicative of *TP53* mutation, in 10–24% of cases^20–22^.

A number of studies have endeavoured to stratify EnOC based on the PROMISE algorithm for endometrial carcinoma classification; however, these have failed to resolve outcome for the majority of EnOC patients with sufficient granularity for clinical implementation, with around 85% of cases classified within the mismatch repair deficient (MMRd) or no specific molecular profile (NSMP) groups which demonstrate equivalent clinical outcome, significantly limiting the utility of PROMISE in EnOC^20–22^.

We recently reported two molecular studies of WT1-negative EnOC: one using whole exome sequencing (WES) to characterise the genomic landscape of EnOC^23^, and one defining molecular subtypes of disease based on hormone receptor expression patterns (HREP-based subtypes)^24^. Both studies identified patient groups with distinct disease-specific and progression-free survival (DSS and PFS). The WES study identified three genomic subtypes across 112 WT1-negative EnOC cases, constructing a stepwise classification taxonomy using *TP53* and *CTNNB1* mutation status, which occur mutually exclusively. The *TP53*m subtype—which accounts for around 25% of cases—was associated with markedly inferior survival, high genomic complexity and frequent CNA events^23^. Conversely, EnOC with wild-type *TP53* (*TP53*wt) and mutation of *CTNNB1* (*TP53*wt/*CTNNB1*m group, ~40% of cases) demonstrated low genomic complexity and few CNA events, and these cases displayed excellent clinical outcome (10-year DSS > 90%). The remaining *TP53*wt/*CTNNB1*wt group, representing around 30% of cases, demonstrated intermediate prognosis with moderate genomic complexity.

The hormone receptor study performed unsupervised analysis of progesterone receptor (PR), oestrogen receptor (ER) and androgen receptor (AR) expression, identifying four HREP-based subtypes driven primarily by PR and ER expression levels: PR + /ER + , PR + /ER−, PR−/ER + and PR−/ER−^24^. Comparative analysis demonstrated that the expression threshold for PR and ER positivity lay at a histoscore of approximately 150 for both markers. The PR + /ER + and PR + /ER− (PR−high) HREP-based subtypes demonstrated excellent prognosis, while the PR−/ER + and PR−/ER− (PR-low) cases demonstrated poor outcome, consistent with earlier reports of favourable prognosis in EnOC demonstrating PR positivity^25,26^.

While it has become clear that EnOC comprises clinically distinct molecular subtypes, the relationship between HREP-based and genomic subtypes is unknown. Here, we investigate the interplay of these two molecular subtyping layers, performing integrated analysis to define the molecular landscape of EnOC with greater granularity and reveal new insights into EnOC biology.

## Results

### Cohort characteristics

**Fig. 1:**
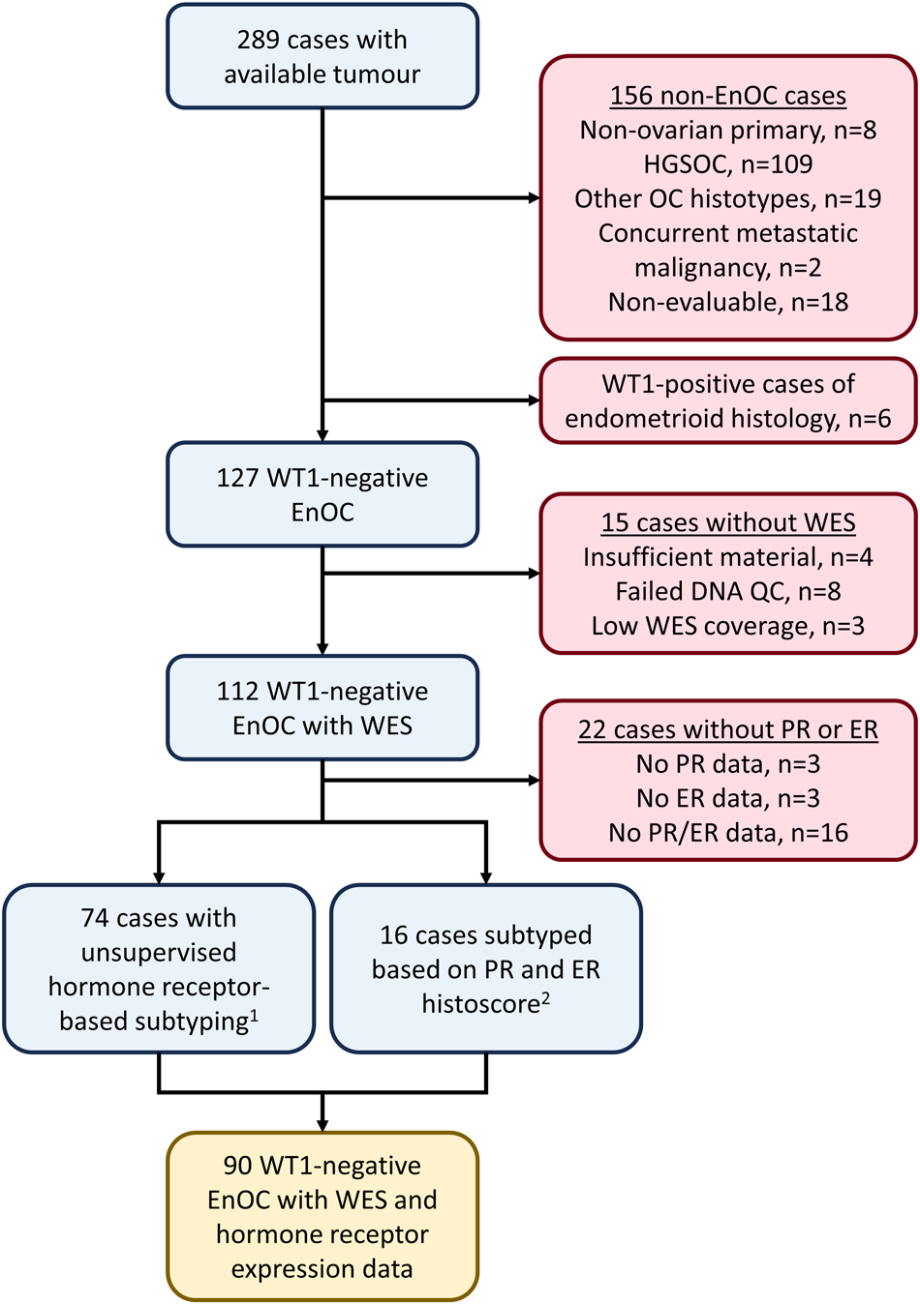
Case flow diagram for endometrioid ovarian carcinoma (EnOC) cases with matched whole exome sequencing (WES) and hormone receptor expression data. ^1^74 EnOC cases with unsupervised hormone receptor subgroup available from the previous study^24^. ^2^16 EnOC cases identified subsequent to the unsupervised subgrouping study of hormone receptor expression patterns with available WES^23^; these cases were classified by available oestrogen and progesterone receptor (ER and PR) immunohistochemistry data, using a histoscore threshold of ≥150. HGS, high grade serous; WT1, Wilms’ tumour 1; QC, quality control; PR, progesterone receptor; ER, oestrogen receptor.

A total of 90 EnOC cases (all WT1 negative) with matched WES and HREP-based subtyping were identified (Fig. 1). Clinical characteristics of the cohort are described in Table 1. The majority of cases were early stage (FIGO I/II) at diagnosis (80.7%, 71 of 88 evaluable cases). 19.0% of cases (16 of 84 evaluable cases) had macroscopic residual disease (RD) following primary debulking surgery.

**Table 1:** Characteristics of 90 endometrioid ovarian carcinoma patients.

		Median (*N*)	Range (%)
Cases	Total	90	
Age	Median years	59.5	28–88
BMI	kg/m^2^	24.7	18.0–44.0
FIGO stage at diagnosis	I	37	42.0
	II^1^	34^2^	38.6
	III	12	13.6
	IV	5	5.7
	NA	2	—
Pathological grade^3^	Grade 1	53	58.9
	Grade 2	13	14.4
	Grade 3	24	26.7
RD following debulking	Zero	68	81.0
	Macroscopic	16	19.0
	NA	6	—
Diagnosis period	1980s	12	13.3
	1990s	37	41.1
	2000 onward	41	45.6
Hormone receptor-based subtype	PR + /ER +	25	27.8
	PR + /ER-	16	17.8
	PR−/ER +	13	14.4
	PR−/ER−	36	40.0
Genomic subtype	*TP53*m	22	24.4
	*TP53*wt/*CTNNB1*wt	29	32.2
	*TP53*wt/*CTNNB1*m	39	43.3
Adjuvant therapy	Single-agent platinum	42	47.2
	Platinum-taxane	15	16.9
	Other platinum combination	5	5.6
	Other chemotherapy	7	7.9
	No chemotherapy	20	22.5
	NA	1	—
Follow-up	Median years	13.1	95% CI 10.5–18.6
5-year DSS	Proportion	74.3%	95% CI 65.6–84.2%
5-year PFS	Proportion	69.8%	95% CI 60.7–80.2%

BMI body mass index, NA not available, RD residual disease, PRprogesterone receptor, ER oestrogen receptor, m mutant, wt wild-type,DSS disease-specific survival, PFS progression-free survival. ^1^Pelvic extension of disease. ^2^17 stage II cases received single-agent platinum chemotherapy, 11 received platinum-taxane chemotherapy, 3 received other cytotoxic regimes, 2 received no adjuvant chemotherapy; treatment information for one case was unavailable. ^3^Using the International Federation of Gynecology and Obstetrics (FIGO) grading system for endometrioid ovarian carcinomas.

Of the total cohort, 22 cases (24.4%) were of the *TP53*m genomic subtype, with 29 (32.2%) and 39 cases (47.2%) of the *TP53*wt/*CTNNB1*wt and *TP53*wt/*CTNNB1*m subtypes, respectively. 49 cases (54.4%) were in the PR-low HREP-based subtypes (36 PR−/ER−, 13 PR−/ER + ) and 41 cases (45.6%) were in the PR-high subtypes (25 PR + /ER + , 16 PR + /ER−).

### Genomic characteristics of hormone receptor-based subtypes

**Fig. 2:**
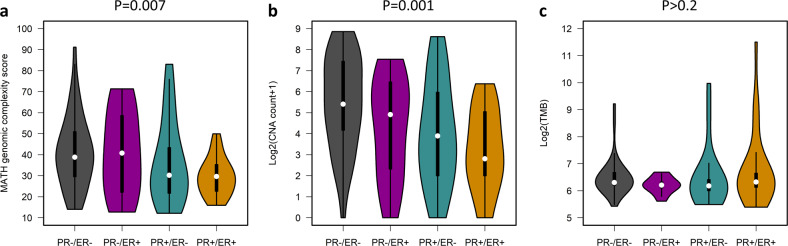
Genomic characteristics of endometrioid ovarian carcinoma subtypes defined by hormone receptor expression patterns. **a** Mutant-allele tumour heterogeneity (MATH) genomic complexity score. **b** Total number of copy number alterations (CNAs). **c** Overall tumour mutation burden (TMB). Labelled *P* values represent comparison of PR-high (PR + /ER + , PR + /ER−) and PR-low (PR−/ER + , PR−/ER−) cases with a two-sided Mann Whitney-U test. For TMB analysis, statistical power to detect a difference between PR-high and PR-low was 0.78 using a two-sided Mann Whitney-U test, assuming exponential distribution and an effect size P(X < Y) of 0.667. PR, progesterone receptor; ER, oestrogen receptor.

PR-high cases demonstrated lower genomic complexity, as quantified by mutant-allele tumour heterogeneity (MATH) genomic complexity score, compared to PR-low cases (median 29.7 vs 38.9, *P* = 0.007) (Fig. 2a), consistent with its low *TP53*m rate (Supplementary Fig. 1). The median MATH scores for the PR + /ER + , PR + /ER−, PR−/ER + and PR−/ER− subtypes were 29.7, 30.2, 40.8 and 38.8, respectively.

The HREP-based subtypes also demonstrated significantly different CNA burden (Fig. 2b); PR-low cases displayed more frequent CNA events compared to PR-high cases (median 37 vs 11, *P* = 0.001). PR−/ER− and PR + /ER + cases demonstrated the greatest and smallest CNA burden, respectively (median 41.5 vs 6 CNA events, *P* < 0.001). There was no significant difference in overall CNA burden between the two PR-high (PR + /ER + vs PR + /ER−, *P* = 0.370) or PR-low subtypes (PR−/ER− vs PR−/ER + , *P* = 0.101). A markedly higher frequency of copy number gain events was noted in the PR−/ER− group specifically (median 25 vs 2, 2.5 and 4 in the PR + /ER + , PR + /ER− and PR−/ER + groups) (Supplementary Figure 2). PR−/ER− cases demonstrated frequent loss of *LNX1* and *SNORA80A*, and gain of *ZNF4*, *STK24* and *CCDC191*; gain of *CDK20* was common in PR-high tumours (Supplementary Tables 1 and 2).

There was no significant difference in tumour mutational burden (TMB) between the HREP-based subtypes (*P* > 0.2) (Fig. 2c); median TMB for the PR + /ER + , PR + /ER-, PR−/ER + and PR−/ER− groups was 80, 72.5, 74 and 79, respectively. The PR-/ER- group demonstrated the highest frequency of mutation in *BRCA1/2*, (19.4%, 7 of 36 cases). This was not statistically significantly higher than in the other groups (5.6%, 3 of 54 cases, *P* = 0.082) (Supplementary Fig. 3); however, statistical power was limited (simulated statistical power of 0.57 to detect a difference of 20% vs 5% in populations of *n* = 36 and *n* = 54).

14 cases (15.6%) displayed mismatch repair (MMR) protein-encoding gene mutations (MMRm) (3 PR-/ER, 3 PR−/ER + , 2 PR + /ER−, 6 PR + /ER + ).

### Correlation of molecular subtyping layers

**Fig. 3:**
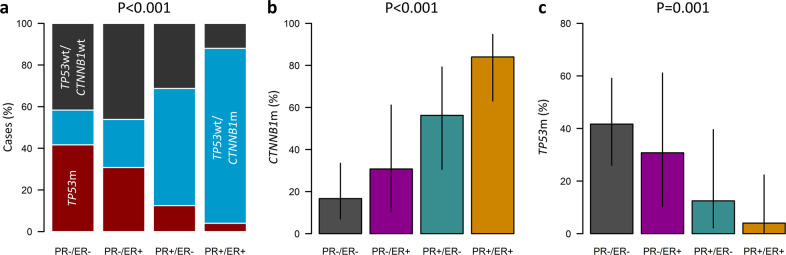
Relationship between endometrioid ovarian carcinoma subtypes. **a** Distribution of genomic subtypes across endometrioid ovarian carcinoma groups defined by hormone receptor expression patterns. **b** Frequency of *CTNNB1* mutation across hormone receptor-based subtypes. **c** Frequency of *TP53* mutation across hormone receptor-based subtypes. Labelled *P* values represent comparison of PR-high (PR + /ER + , PR + /ER−) (*n* = 41) and PR-low (PR-/ER + , PR−/ER−) cases (*n* = 49) using Chi-squared test. For **b** and **c**, vertical lines represent the 95% confidence intervals for true proportion. PR, progesterone receptor; ER, oestrogen receptor; m, mutant; wt, wild-type.

There was marked overlap between HREP-based and genomic subtyping layers (*P* < 0.001) (Fig. 3a, Supplementary Table 3): PR-high cases were predominantly of the *TP53*wt/*CTNNB1*m subgroup (73.2%, 30 of 41 cases vs 18.4%, 9 of 49 PR-low cases, *P* < 0.001), while the *TP53*m was more common in PR-low cases (38.8%, 19 of 49 cases vs 7.3%, 3 of 41 PR-high cases, *P* = 0.001). Specifically, the PR + /ER + subtype was overwhelmingly of the *TP53*wt/*CTNNB1*m genomic subtype (84.0%, 21 of 25) (Fig. 3b), with the lowest *TP53*m rate (4.0%, 1 of 25) (Fig. 3c). Conversely, the PR-/ER- group had the fewest *TP53*wt/*CTNNB1*m cases (16.7%, 6 of 36 cases), and the largest proportion of *TP53*m (41.7%, 15 of 36 cases).

There was no association between HREP-based subtyping and mutation status of *ARID1A*, *PTEN* or *KRAS* (Supplementary Table 4). PR-high cases demonstrated a high rate of *PIK3CA* mutation (63.4%, 26 of 41 vs 38.8%, 19 of 49 in PR-low, *P* = 0.034), but this was not statistically significant following adjustment for multiplicity of testing (Bonferroni-adjusted *P* = 0.137) (Supplementary Table 4).

### Hormone receptor expression levels in genomic subtypes of endometrioid ovarian carcinoma

**Fig. 4:**
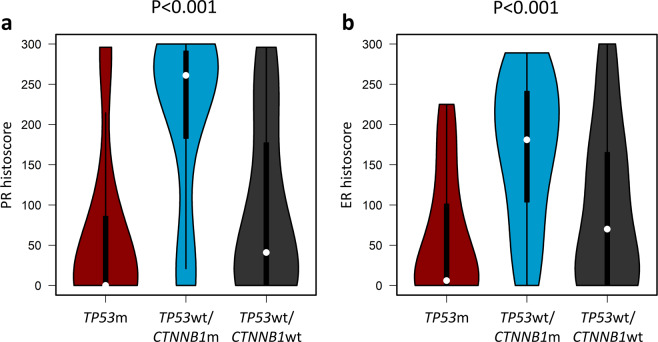
Hormone receptor expression across genomic subtypes of endometrioid ovarian carcinoma. **a** Histoscore for progesterone receptor (PR) expression. Labelled *P* value denotes comparison of *TP53*m (*n* = 22) and *TP53*wt/*CTNNB1*m (*n* = 39) cases with a two-sided Mann Whitney-U test; additionally, *TP53*m vs *TP53*wt/*CTNNB1*wt (*n* = 29) *P* = 0.119 and *TP53*wt/*CTNNB1*wt vs *TP53*wt/*CTNNB1*m *P* < 0.001. **b** Histoscore for oestrogen receptor (ER) expression. Labelled *P* value denotes comparison of *TP53*m and *TP53*wt/*CTNNB1*m cases with a two-sided Mann Whitney-U test; additionally, *TP53*m vs *TP53*wt/*CTNNB1*wt *P* = 0.164 and *TP53*wt/*CTNNB1*wt vs *TP53*wt/*CTNNB1*m *P* = 0.002. m, mutant; wt, wild-type.

*TP53*wt/*CTNNB1*m cases expressed high levels of PR (median PR histoscore = 261) (Fig. 4a), while *TP53*m cases were predominantly PR negative (median PR histoscore = 0, *P* < 0.001 vs the *TP53*wt/*CTNNB1*m group). The *TP53*wt/*CTNNB1*wt subtype also demonstrated significantly lower PR levels than the *TP53*wt/*CTNNB1*m cases (median PR histoscore = 41, *P* < 0.001). The *TP53*wt/*CTNNB1*m and *TP53*m subtypes demonstrated the highest and lowest levels of ER expression, respectively (median ER histoscore 181 vs 6, *P* < 0.001) (Fig. 4b).

PR and ER histoscores showed modest but statistically significant anti-correlation with CNA burden (rho = −0.32, *P* = 0.002 and −0.39, *P* < 0.001) and MATH genomic complexity score (rho = −0.23, *P* = 0.026 and −0.27, *P* = 0.011).

### Hormone receptor expression-based subtype is independently associated with favourable outcome

Multivariable survival analysis of genomic and HREP-based subtypes—accounting for stage at diagnosis, pathological grade, patient age and presence of macroscopic RD following debulking—identified the PR-high groups as independently associated with prolonged survival (DSS HR for PR-high = 0.16, 95% CI 0.04–0.71; PFS HR for PR-high = 0.29, 95% CI 0.09–0.99) (Supplementary Tables 5 and 6).

### *TP53* mutation defines highest risk cases in PR-low endometrioid ovarian carcinomas

**Fig. 5:**
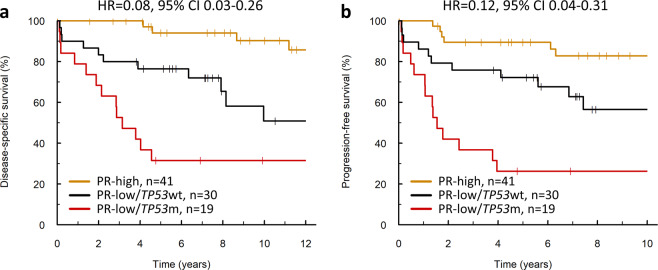
Clinical outcome of endometrioid ovarian carcinoma cases defined by combined HREP-based subtyping and *TP53* mutation status. **a** Disease-specific survival. Labelled hazard ratio (HR) represents comparison of PR-high with PR-low/*TP53*m group (*P* < 0.0001); additionally, HR for PR-high vs PR-low/*TP53*wt = 0.21, 95% CI 0.07–0.67 and HR for PR-low/*TP53*m vs PR-low/*TP53*wt = 2.56, 95% CI 1.14–5.75. **b** Progression-free survival. Labelled HR represents comparison of PR-high with PR-low/*TP53*m group (*P* < 0.0001); additionally, HR for PR-high vs PR-low/*TP53*wt = 0.34, 95% CI 0.13–0.92 and HR for PR-low/*TP53*m vs PR-low/*TP53*wt = 2.93, 95% CI 1.32–6.51. PR, progesterone receptor; m, mutant; wt, wild-type.

The 10-year DSS in the PR-high and PR-low cases was 90.3% and 44.0%, respectively. Incorporation of *TP53*m status delineated outcome within the PR-low group; PR-low cases with *TP53*m (PR-low/*TP53*m) demonstrated poor outcome compared to their *TP53*wt (PR-low/*TP53*wt) counterparts (HR for DSS = 2.56, 95% CI 1.14–5.75, *P* = 0.022; HR for PFS = 2.93, 95% CI 1.32–6.51, *P* = 0.008) and excellent outcome resolution between PR-high and PR-low/*TP53*m cases (HR for DSS = 0.08, 95% CI 0.03–0.26, *P* < 0.0001 and HR for PFS = 0.12, 95% CI 0.04–0.31, *P* < 0.0001) (Fig. 5).

Leave-one-out jackknifing demonstrated negligible impact of outlier cases within this model (PR-high vs PR-low/*TP53*m: median DSS HR = 0.08, σ^2^ = 2.6 × 10^−5^ median PFS HR = 0.12, σ^2^ = 3.8 × 10^−5^) (Supplementary Fig. 4). Resampling analysis to simulate non-identical EnOC cohorts (1000 simulated cohorts of *N* = 90 by resampling with replacement) demonstrated stability of the observed survival phenotypes (PR-high vs PR-low/*TP53*m: median DSS HR = 0.07, σ^2^ = 0.003; median PFS HR = 0.11, σ^2^ = 0.004) (Supplementary Fig. 5); 99.6% and 98.9% of simulations yielded an HR < 0.30 for DSS and PFS, respectively.

The three-tier integrated classification taxonomy significantly delineated outcome specifically in the context of stage II disease (DSS HR for PR-high vs PR-low/*TP53*m = 0.06 and PFS HR for PR-high vs PR-low/*TP53*m = 0.14; *P* = 0.019 and *P* = 0.013).

### Features of integrated EnOC subtypes and recapitulation with immunohistochemistry

Compared to the PR-low/*TP53*wt cases, the PR-low/*TP53*m group demonstrated more frequent advanced stage at diagnosis (52.6%, 10 of 19 cases vs 13.3%, 4 of 30 cases, *P* = 0.011) and macroscopic RD following debulking (50.0%, 9 of 18 evaluable cases vs 14.3%, 4 of 28 evaluable cases, *P* = 0.022) (Supplementary Table 7). Moreover, they demonstrated greater genomic complexity by MATH score (median 54.7 vs 32.7, *P* < 0.001) and greater CNA burden (median 98 vs 24 CNA events, *P* = 0.001) (Supplementary Fig. 6a). Indeed, the clinicopathological features of PR-low/*TP53*wt cases were similar to the PR-high group, showing no difference in stage at diagnosis or RD status (*P* = 0.691 and *P* = 0.446) (Supplementary Table 7). There was no difference in CNA burden or MATH genomic complexity score between PR-high and PR-low/*TP53*wt cases (*P* = 0.623 and *P* = 0.162) (Supplementary Fig. 6). PR-low/*TP53*wt cases demonstrated significantly poorer outcome compared to PR-high cases despite similar clinicopathological features (DSS HR for PR-high vs PR-low/*TP53*wt = 0.21, 95% CI 0.07–0.67) (Fig. 5).

A simple classification algorithm utilising IHC for PR (histoscore < 150 vs ≥150) and p53 (wild-type pattern vs aberrant expression) recapitulated these subtypes and their survival profiles (Supplementary Table 8 and Supplementary Fig. 7).

### Genomic events suggest specific therapeutic vulnerabilities in high-risk EnOC

PR-low/*TP53*m cases represented the highest risk EnOC patient group (10-year DSS 31.6%); cases with PR-low/*TP53*wt tumours also represented patients for which new treatment options are needed to improve survival (10-year DSS 50.9%). 90.0% of the PR-low/*TP53*wt group (27 of 30 cases) demonstrated mutations in *KRAS* (12 cases), *PTEN* (7 cases), *PIK3CA* (15 cases) or *BRCA1/2* (2 cases). 47.4% of the PR-low/*TP53*wt group demonstrated mutations in *KRAS* (2 cases), *PTEN* (1 case), *PIK3CA* (4 cases) or *BRCA1/2* (6 cases).

High-risk EnOC cases demonstrated frequent missense mutation in *SOX8* (26.3% in PR-low/*TP53*m; 10.0%, 3 of 30 in PR-low/*TP53*wt), a transcription factor associated with the WNT/β-catenin pathway.

## Discussion

EnOC is a relatively under-investigated type of ovarian cancer; recently, molecular subtypes of EnOC have been described at the genomic and protein expression level—by *TP53* and *CTNNB1* mutation status, and by patterns of hormone receptor expression—and these have been associated with distinct clinicopathological features and survival outcome^23,24^. Here we dissect the relationship between genomic and HREP-based subtypes, demonstrating marked overlap between these two subtyping layers.

We show that the *TP53*wt/*CTNNB1*m genomic subtype overwhelmingly expresses high levels of PR and ER, while *TP53*m is associated with low expression of these markers; accordingly, the PR-high HREP-based subtypes are frequently of the *TP53*wt/*CTNNB1*m genomic subtype, while the PR-low cases are more frequently of the *TP53*m and *TP53*wt/*CTNNB1*wt genomic subtypes.

We also demonstrate that the HREP-based subtypes display significant differences in their global genomic landscape: PR-high cases demonstrate low CNA burden, while CNA events are more common in PR-low cases. In particular, the PR-/ER- subtype displayed the highest CNA burden, with markedly more frequent copy number gain events compared to the other subtypes. The low rate of *TP53*m in PR-high cases may well underpin the reduced CNA burden in this population.

PR-high EnOC also represents those with the lowest MATH score, a quantitative score calculated using global mutant VAF distributions to infer tumour genomic complexity^27^. The higher MATH score in PR-low patients suggests greater intratumor heterogeneity in these cases^28^, with multiple subclonal populations harbouring distinct mutational landscapes. This heterogeneity may well contribute to treatment failure, with greater diversity increasing the likelihood of an EnOC cancer cell population surviving primary chemotherapy and seeding subsequent chemoresistant relapse. Higher genomic complexity may therefore contribute to the poor outcome experienced by this patient group. These data suggest that PR-low EnOC represent a subtype reminiscent of HGSOC, demonstrating greater genomic complexity, more frequent CNA events, higher rates of *TP53*m, and frequent advanced stage at diagnosis with overall poor clinical outcome^3,7,8^.

Multivariable analysis of molecular subtyping layers, accounting for clinicopathological factors, demonstrated favourable outcome in the PR-high subtypes independent of correlation with *CTNNB1*m and anti-correlation with *TP53*m. This suggests prospective classification of newly diagnosed EnOC into HREP-based subtypes using IHC for PR and ER may represent a meaningful way to identify EnOC patients with high- and low-risk disease. The threshold for ER and PR positivity in this taxonomy lay at a histoscore of approximately 150, and assessment of nuclear marker expression by histoscore is already routinely performed as part of the diagnostic pathology pipeline in many centres^29–31^. Indeed, pathological grade demonstrated no meaningful or significant association with outcome in this analysis (HR = 0.99 for DSS), suggesting that PR IHC—a more objective and quantifiable marker—outperforms pathological grade with regard to prognostication, which is assessed subjectively. This is consistent with a recent report demonstrating the limited utility of pathological grading in a large cohort of EnOC^21^.

While the PR-low groups demonstrate significantly poorer outcome compared to their PR-high counterparts, we show that *TP53*m is a useful additional discriminator within the PR-low group. PR-low/*TP53*m cases harboured greatest CNA burden, were more frequently advanced stage and incompletely surgically resected, and demonstrated dismal prognosis. These cases represent a patient group for which new treatment options are most urgently needed to improve survival. By contrast, PR-low/*TP53*wt cases demonstrated intermediate prognosis, despite clinicopathological characteristics more akin to those of PR-high cases (frequent early stage at diagnosis and complete macroscopic resection).

Together, these data suggest that combined use of HREP-based and genomic data—particularly PR expression and *TP53*m status—can delineate EnOC patient outcome with greater granularity. An immunohistochemical algorithm, utilising IHC for p53 and β-catenin—the gene products of *TP53* and *CTNNB1*—has been suggested as a surrogate for identifying the reported genomic subtypes of EnOC in routine diagnostic pathology^23^. Given the combined utility of HREP-based subtypes and *TP53*m status described here, coupled with the reported sensitivity and specificity issues of β-catenin IHC as a surrogate for *CTNNB1*m^23,32,33^ and the high correlation between PR expression and *CTNNB1*m, an algorithm utilising IHC for PR/ER and p53 may provide a more practical approach by which to prospectively classify newly diagnosed EnOC. Indeed, we demonstrate that a simple two-marker classifier using PR and p53 IHC can identify high- and low-risk EnOC patient groups. IHC for p53, ER and PR is already routinely performed as part of the pathological diagnostic pipeline across many centres, and identification of subtypes in this way is therefore readily implementable with relatively low cost and little change in current practice. Validation of this classification approach in an independent cohort is required prior to its potential implementation.

By comparison, efforts to apply the endometrial carcinoma molecular classifier—the PROMISE algorithm—have not identified a sizeable proportion of excellent prognosis EnOC patients^20–22^; *POLE*-mutated tumours are reportedly associated with around 90% 10-year overall patient survival, but account for only 3.5% of cases, limiting implementation of *POLE* as a prognostic marker. Moreover, two of the four subtypes identified by this algorithm—the MMRd and NSMP groups—account for around 85% of cases and demonstrate equivalent clinical behaviour^21^, limiting the utility of this classification system.

The identification of patients at higher risk of disease relapse is arguably the highest priority for studies investigating patient stratification. While identification of low-risk patients for de-escalation of therapy may spare some patients the adverse effects of systemic chemotherapy, identifying those who derive most benefit from systemic cytotoxic agents and novel treatment options represents an opportunity to rescue the greatest number of life years. The PR-low/*TP53*m and PR-low/*TP53*wt groups represent cases for which new treatment options are needed to improve survival. The genomic profiles of PR-low/*TP53*wt cases demonstrated frequent *KRAS*, *PTEN* and *PIK3CA* mutations, highlighting MAPK and PIK3-AKT pathway inhibitors as candidate agents of potential therapeutic utility in this patient group. Indeed, inhibition of the MAPK pathway has recently demonstrated favourable efficacy in low grade serous ovarian carcinoma^34^, which is known to demonstrate frequent *KRAS* mutation^35^. By contrast, PR-low/*TP53*m tumours rarely demonstrate such mutations; however, around one third (31.6%, 6 of 19) display mutation of *BRCA1/2*, suggesting exploration of poly-(ADP-ribose) polymerase (PARP) inhibitor efficacy in this population is warranted.

PR-low/*TP53*m cases also demonstrated a high rate of missense mutation in *SOX8*, encoding a transcription factor implicated in the regulation of WTN/β-catenin signalling^36^; while the precise signalling consequences of such mutations are not yet well defined, these data suggest that inhibitors of the WNT pathway may represent a candidate therapeutic strategy in these cases, with few other recurrent targetable events identified in this patient population. The marked clinical and molecular heterogeneity demonstrated by EnOC highlights the critical need for rationally designed trials to account for molecular subtypes of disease when investigating new agents for EnOC.

Endocrine therapy has been suggested as a potential treatment strategy for EnOC^37^. In particular, de-escalation of primary treatment from chemotherapy to endocrine therapy has been suggested as a potential approach for patients with stage II EnOC with zero macroscopic RD who would otherwise receive cytotoxic chemotherapy^24^, which has both significant toxicities and resource implications. By contrast, endocrine therapy is well tolerated, does not need to be delivered at a specialist centre, and is low in cost^38^. Indeed, there has been growing interest in the use of these agents in ovarian cancer as both treatment for relapsed HGSOC^39,40^ and maintenance therapy for LGSOC^41^, which is typically ER-positive^25^. In relapsed HGSOC, high ER histoscore and prolonged treatment-free interval have been associated with greater efficacy of endocrine agents^40^. We demonstrate that *TP53*wt/*CTNNB1*m EnOC are overwhelmingly positive for ER and PR, suggesting this subtype of patients may represent those most likely to benefit from such strategies. Given the high rate of early stage disease (FIGO stage I/II) and complete surgical resection in this subtype, efforts aimed at assessing the feasibility of adjuvant chemotherapy de-escalation should focus on this patient population. Endocrine agents represent a potential alternative treatment strategy worthy of investigation in this low-risk patient group.

This study makes use of a unique, robustly defined EnOC cohort with matched genomic and hormone reception expression data; while this affords the opportunity to perform integrated analyses that were not previously possible in this disease type, we recognise a number study limitations. To our knowledge, no other dataset of this type is available with matched genomic characterisation and PR/ER expression data; as such, we were unable to utilise an independent dataset to validate our findings. We have, however, employed leave-one-out jackknifing and permutative resampling approaches to assess the impact of outliers and simulate the effect size of our integrated classification model in non-identical EnOC cohorts. We also recognise our application of multiple independent statistical analyses, though we have applied multiplicity correction where appropriate and note that the survival associations we report are highly statistically significant (*P* < 0.0001 in our final DSS model; PR-high vs PR-low/*TP53*m).

Together, these data shed light on the overlay and interplay of molecular subtyping layers in EnOC and highlight the distinct clinical and molecular phenotypes displayed by these patient groups. Patients with PR-low EnOC harbouring *TP53*m represent the highest risk group, with frequent disease relapse; these patients experience poor prognosis, in stark contrast to those with PR-high tumours, which are typically *TP53*wt, frequently harbour *CTNNB1*m and are associated with excellent patient survival. The low PR and ER expression in *TP53*m cases suggests that they are unlikely to benefit from endocrine therapy, while PR-high cases may represent the best candidates for these agents.

Future work should seek to validate the clinical impact of these groups in an independent cohort of EnOC and investigate new treatment options for high risk patients. Rigorous contemporary pathological review, with routine use of WT1 IHC to exclude pseudo-endometrioid HGSOC, will be essential for robust validation. Inhibitors of the MAPK and PI3K-AKT pathways represent promising candidates for improving outcome in PR-low/*TP53*wt EnOC. By contrast, the PR-low/*TP53*m group demonstrates fewer clinically actionable recurrent mutations. PARP inhibitors represent promising agents for *BRCA1/2* mutant patients within this highest risk population; inhibitors of the WNT/β-catenin pathway represent further agents worthy of investigation in this subtype given the high frequency of mutation in *SOX8*. IHC for PR and p53—both of which are already performed within the routine diagnostic pipeline for ovarian tumours—represents a readily implementable approach for identifying high- and low-risk EnOC patient groups.

## Methods

### Cohort identification and clinical annotation

A total of 112 EnOC cases underwent WES following pathological review as previously described^23^, of which 90 cases had matching PR and ER expression data (Fig. 1). Of 505 identified ovarian cancer cases with a documented EnOC diagnosis, tumour material was available for 289 cases and pathology review was performed by an expert gynecological pathologist (CSH), which included WT1 IHC for every case. WT1 IHC was performed with 1:1000 anti-human WT1 monoclonal mouse antibody clone 6F-H2 on the Leica BOND III Autostainer using IHC protocol F. WT1-positive cases, tumours of non-ovarian primary, and ovarian cancer histotypes other than EnOC were excluded (Fig. 1). Ethical approval for the study was obtained from NHS Lothian Research and Development (reference 2007/W/ON/29) and Lothian NRS BioResource (reference 15/ES/0094-SR494). All relevant ethical regulations have been complied with, including the need for written informed consent where required. Clinicopathological characteristics at diagnosis, treatment details and outcome data were retrieved from the Edinburgh Ovarian Cancer Database^4^. DSS and PFS were calculated from date of pathologically confirmed diagnosis; PFS was defined as time to radiologically confirmed recurrence or progression, or death from EnOC.

### Genomic characterisation

Genomic data were available from the previous WES study of 112 EnOC cases^23^. Formalin-fixed paraffin-embedded (FFPE) tumour material was macrodissected using haematoxylin and eosin-stained slides as a guide, marked to identify tumour areas by an expert pathologist (CSH). DNA was extracted using the Qiagen QIAamp DNA FFPE Tissue Kit and Deparaffinization Solution. Tumour DNA underwent WES to a mean per-sample on-target depth of 89.5X using the Illumina TruSeq Exome Library Prep kit on an Illumina NextSeq 550, and processed as previously described using the bcbio-nextgen integrated bioinformatic pipeline (v1.0.9)^23^.

Briefly, reads were aligned to GRCh38 with BWA v0.7.17, duplicates were marked, base quality scores were recalibrated, and variant calling was performed using a majority vote system between three variant callers (Mutect2^42^, VarDict^43^ and Freebayes^44^). Variants were filtered for FFPE and oxidation artefacts, low variant allele frequency (VAF) (<10%), low variant coverage (<20X), and for common variants using the ExAC (ExAC.0.3.GRCh38) and 1000 Genomes (Version phase 1 SNP and InDel) reference datasets. Variants of known functional significance were flagged using the ClinVar database, and remaining variants were annotated using PolyPhen and SIFT prediction tools to filter likely non-functional variants.

TMB was calculated as the mutation count per sample, following the above-described filtering steps. CNA events were identified using the GeneCN pipeline in bio-DB-HTS version 2.10. Genomic complexity was quantified by MATH score, calculated from per-sample VAF density distribution using the inferHeterogeneity function in the maftools R package^27^.

### Hormone receptor expression and p53 immunohistochemistry

ER and PR expression data were available for 90 of the 112 EnOC cases characterised by WES (Fig. 1)^24^. IHC for PR and ER was performed on human tissue microarrays comprising three 0.8 mm cores per case, as previously described^24^. IHC used 1:50 mouse anti-PR antibody M3569 clone PgR-636 and 1:50 rabbit anti-ER antibody M3643 clone EP1; PR and ER IHC was performed on the Leica BOND III Autostainer and expression was assessed by histoscore, a quantitative nuclear expression score incorporating staining intensity and proportion of positive tumour nuclei^31^. HREP-based subtype was available for 74 cases from the unsupervised subtyping study^24^;16 further cases were classified based on their PR and ER histoscore using a threshold of histoscore ≥150 for positivity, as indicated by the unsupervised subtyping study^24^ (Fig. 1).

p53 IHC data were available for 87 of the 90 cases^23^. IHC was performed on the Leica BOND III Autostainer using a 1:50 dilution of p53 antibody (clone DO-7, DAKO). Wild-type pattern was defined as variable nuclear staining intensity, while aberrant staining was defined as strong diffuse nuclear positivity (aberrant positive) or complete absence of nuclear staining (aberrant null).

### Statistical analysis

Statistical analyses were performed using R 4.0.3. Categorical variables were compared using Fisher’s exact test or the Chi-square test, as appropriate; continuous data were compared using the Mann Whitney-U test. Spearman’s rank coefficient was used to assess correlation between continuous variables. Comparisons of survival were made using Cox proportional hazards models within the Survival package, presented as hazard ratios (HRs) and corresponding 95% confidence intervals (95% CIs). Median follow-up time was calculated using the reverse Kaplan–Meier method. Correction for multiplicity of testing was applied using the Bonferroni method where specified.

The impact of outlier cases on the final integrated classification taxonomy was assessed using a leave-one-out jackknife approach; survival analysis was repeated for all possible iterations of a single missing sample. The outcome of the jackknifing analysis is presented as the median HR of all iterations, alongside the HR variance (σ^2^). Applicability of the final integrated classification taxonomy to non-identical EnOC cohorts was assessed by the generation of 1000 simulated cohorts via resampling with replacement (1000 iterations of *N* = 90 resampling) and analysis of the HR distribution across these simulated datasets; results are reported as the median HR, HR variance and the proportion of iterations demonstrating a large effect size (HR < 0.30).

### Reporting summary

Further information on research design is available in the Nature Research Reporting Summary linked to this article.

## Supplementary information

Supplementary Information

Reporting Summary

## Data Availability

The genomic data described in this manuscript are available via the European Genome-phenome Archive (accession EGAS00001004366). We are happy to provide the hormone receptor expression data described here, upon reasonable request.
