# Supplementary material for: Integrated molecular characterisation of endometrioid ovarian carcinoma identifies opportunities for stratification
Source: NPJ Precis Oncol. 2021 Jun 2;5:47. doi: 10.1038/s41698-021-00187-y (PMC8172925; doi:10.1038/s41698-021-00187-y)
Supplement: Supplementary file 1 — Supplementary Information [file 41698_2021_187_MOESM1_ESM.pdf]

SUPPLEMENTARY INFORMATION

Integrated molecular characterisation of endometrioid ovarian carcinoma identifies opportunities for stratification

Robert L Hollis[1], Barbara Stanley[1,2], John P Thomson[1], Michael Churchman[1], Ian Croy[1], Tzyvia Rye[1], Clare Bartos[1], Fiona Nussey[3], Melanie Mackean[3], Alison M Meynert[4], Colin A Semple[4], Charlie Gourley[1*] and C. Simon Herrington[1*]

[1]Nicola Murray Centre for Ovarian Cancer Research, Cancer Research UK Edinburgh Centre, MRC Institute of Genetics and Cancer, University of Edinburgh, UK

[2]Beatson West of Scotland Cancer Centre and University of Glasgow, Glasgow, UK

[3]Edinburgh Cancer Centre, Western General Hospital, Edinburgh, UK.

[4]MRC Human Genetics Unit, MRC Institute of Genetics and Cancer, University of Edinburgh, UK

[*]these authors contributed equally

SUPPLEMENTARY FIGURES

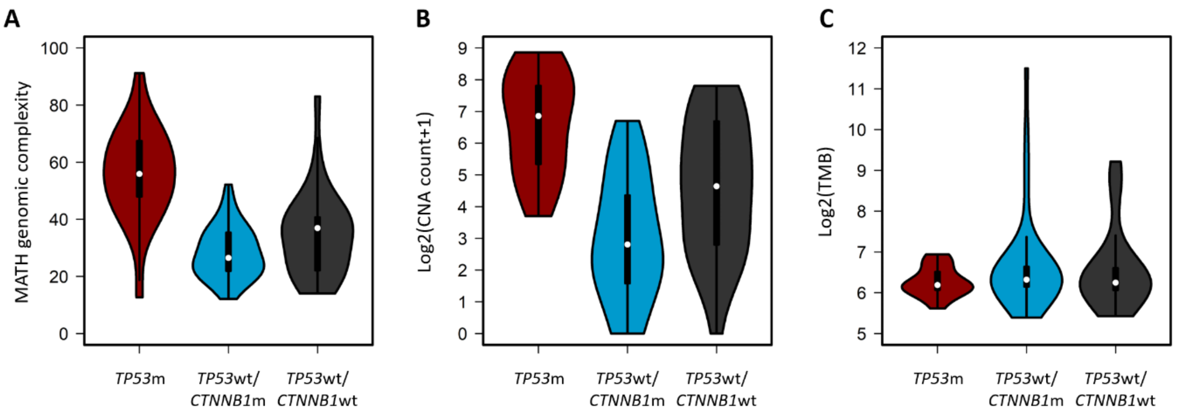

Supplementary Figure 1. Global genomic characteristics of previously reported EnOC subtypes. (A) Mutant-allele tumor heterogeneity (MATH) genomic complexity score. (B) Total number of copy number alterations (CNAs). (C) Overall tumor mutation burden (TMB). PR, progesterone receptor; ER, estrogen receptor.

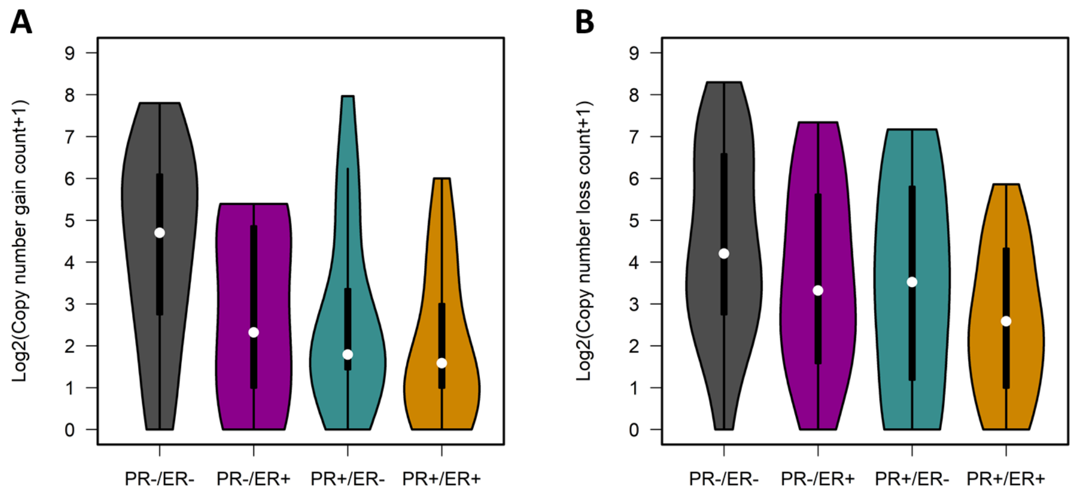

Supplementary Figure 2. Number of (A) copy number gain events and (B) copy number loss events across hormone receptor-based endometrioid ovarian carcinoma subtypes. PR, progesterone receptor; ER, estrogen receptor.

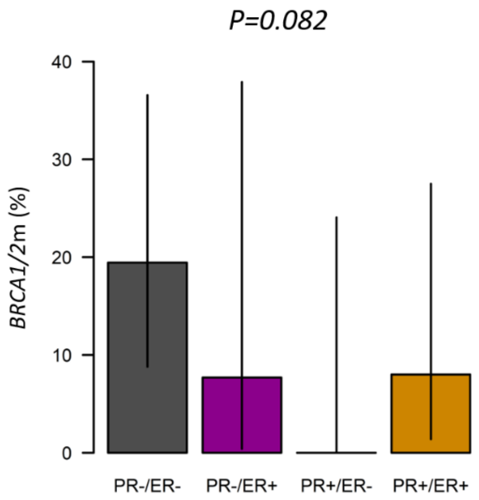

Supplementary Figure 3. Frequency of *BRCA1/2* mutation across hormone receptor-based subtypes in endometrioid ovarian carcinoma. Vertical lines represent the 95% confidence interval of the true proportion. Labelled P value represents the comparison of PR-/ER- cases (n=36) versus all other cases (n=54). Simulated statistical power to detect a significant difference was approximately 0.57 (for 20% vs 5% of n=36 vs n=54 at an alpha of 0.05 for a two-sided Fisher's exact test). PR, progesterone receptor; ER, estrogen receptor; m, mutant.

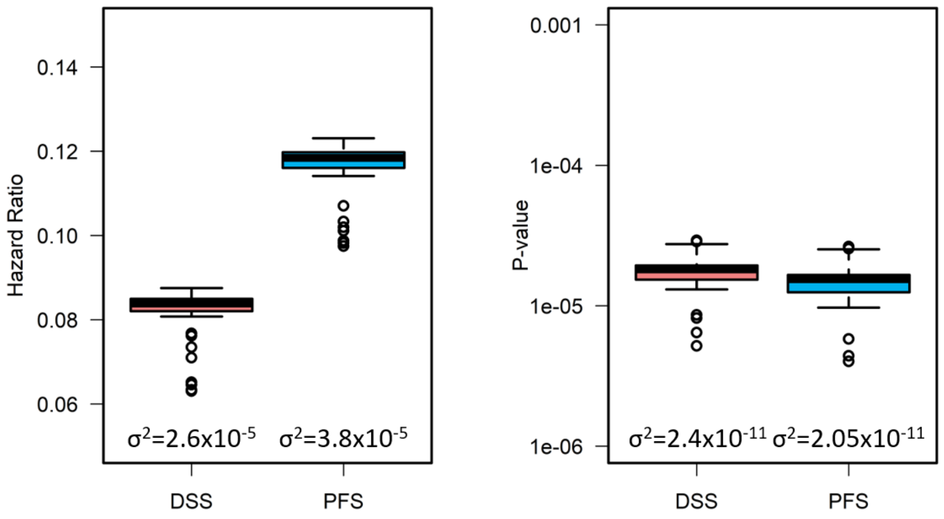

Supplementary Figure 4. Summary of leave-one-out jackknife analysis. (A) Hazard ratios from each iteration of jackknifing comparing PR-high cases vs PR-low/TP53m cases. (B) P-values from each iteration of jackknifing comparing PR-high cases vs PR-low/TP53m cases. Boxes represent 1st to 3rd

quartile, with the median labelled as the central line; whiskers extend to the data range from 1st and 3rd quartile +/-1.5 times the interquartile range. DSS, disease-specific survival. PFS, progression-free survival.

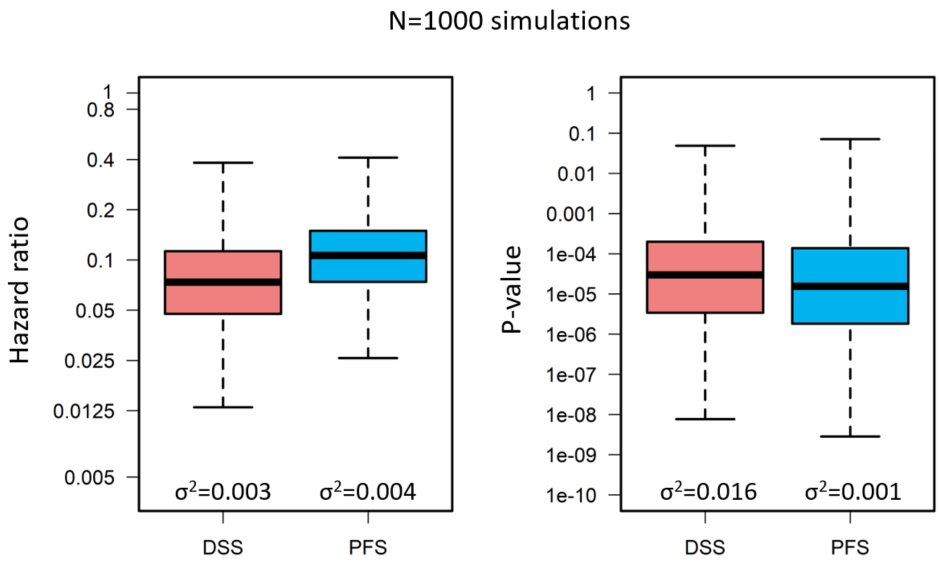

Supplementary Figure 5. Summary of survival differences in 1000 simulated n=90 EnOC cohorts generated by resampling with replacement. (A) Hazard ratios from simulated cohort comparing PR-high cases vs PR-low/TP53m cases. (B) P-values from each simulated cohort comparing PR-high cases vs PR-low/TP53m cases. Boxes represent 1st to 3rd quartile, with the median labelled as the central line; whiskers extend to the data range from 1st and 3rd quartile +/-1.5 times the interquartile range. DSS, disease-specific survival. PFS, progression-free survival.

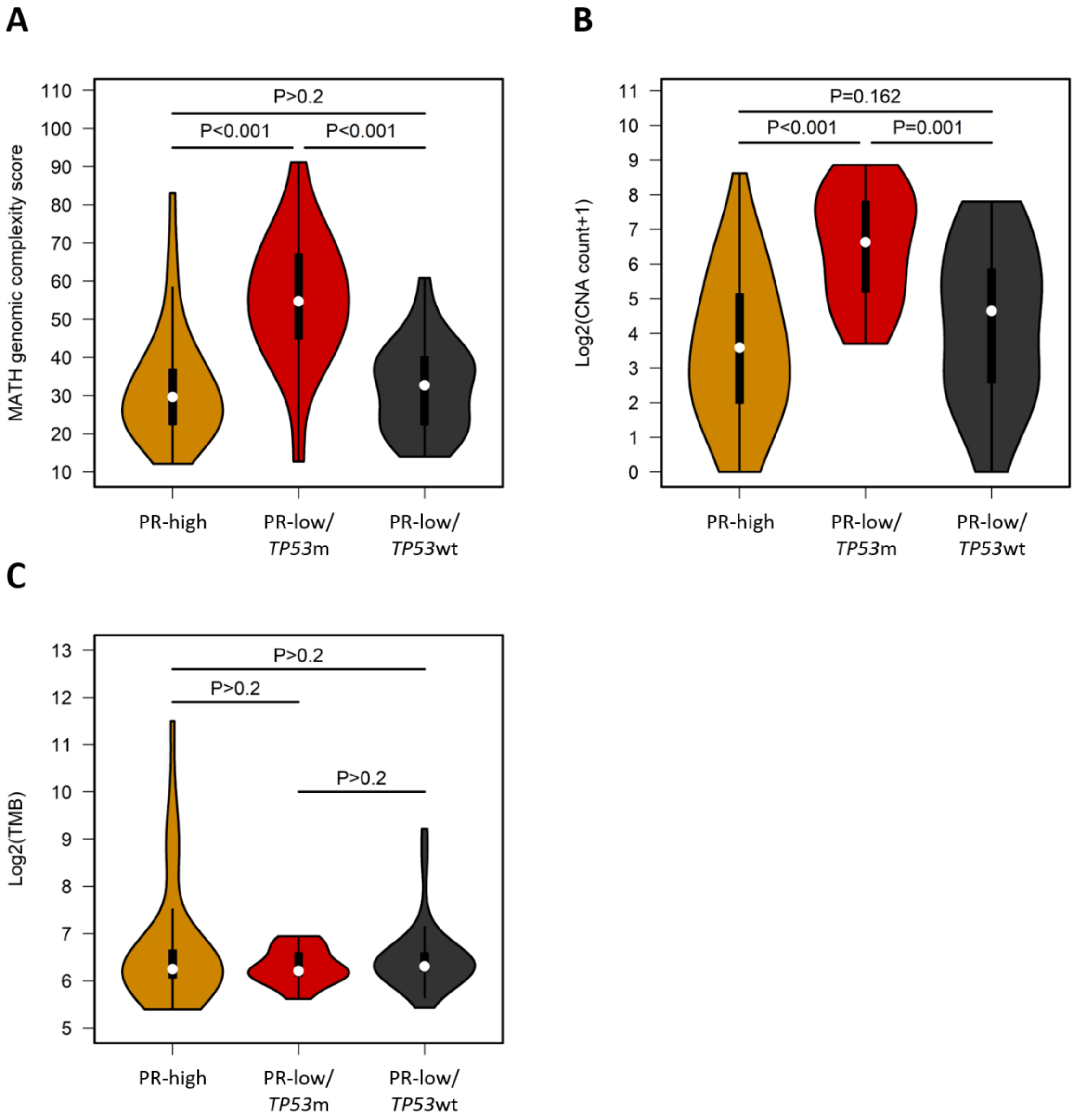

Supplementary Figure 6. Genomic characteristics of endometrioid ovarian carcinoma subtypes defined by combined hormone receptor expression-based subtype and *TP53* mutation status. (A) Mutant-allele tumor heterogeneity (MATH) genomic complexity score, (B) copy number alteration (CNA) count and (C) tumor mutational burden (TMB). All comparisons are made using two-sided Mann Whitney-U tests. For TMB analysis, statistical power to detect a difference between PR-high and PR-low/*TP53*-wildtype was 0.67 using a two-sided Mann Whitney-U test, assuming exponential distribution and an effect size P(X<Y) of 0.667. PR, progesterone receptor; m, mutant.

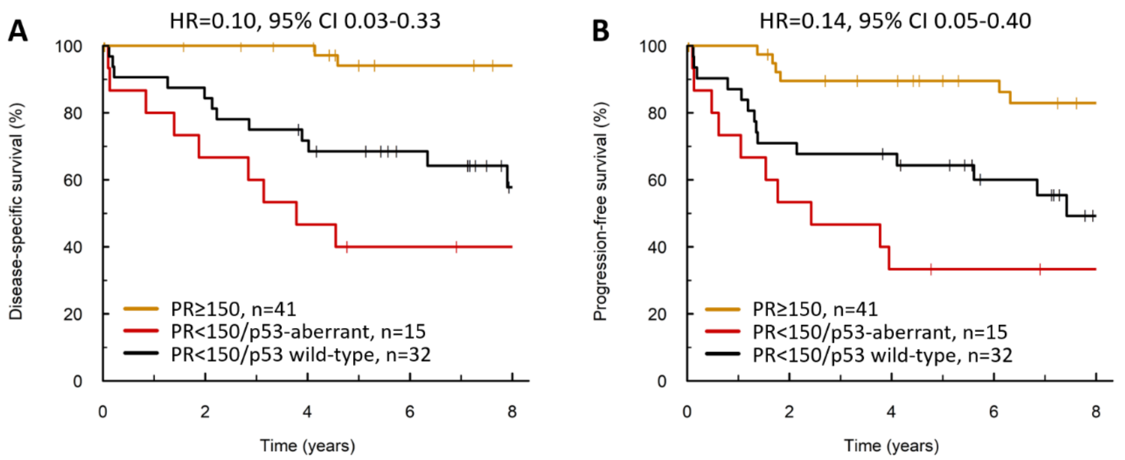

Supplementary Figure 7. Clinical outcome of endometrioid ovarian carcinoma cases defined by immunohistochemistry for progesterone receptor (PR) and p53. (A) Disease-specific survival. Labelled hazard ratio (HR) represents comparison of PR≥150 with PR<150/p35-aberrant group. (B) Progression-free survival. Labelled HR represents comparison of PR≥150 with PR<150/p35-aberrant group. PR≥150, PR histoscore ≥150; PR<150, PR histoscore <150; p53-aberrant, aberrant p53 protein expression profile; p53 wild-type, wild-type p53 protein expression pattern.

SUPPLEMENTARY TABLES

Supplementary Table 1. Common copy number loss events in endometrioid ovarian carcinoma

	Chromosome	N	%	PR+/ER+		PR+/ER-		PR-/ER+		PR-/ER-	
				N	%	N	%	N	%	N	%
PKNOX1	21	36	40.0%	9	36.0%	5	31.3%	5	38.5%	17	47.2%
CEP68	2	21	23.3%	6	24.0%	3	18.8%	4	30.8%	8	22.2%
SNORA80A	21	18	20.0%	2	8.0%	3	18.8%	2	15.4%	11	30.6%
TBC1D1	4	17	18.9%	2	8.0%	4	25.0%	1	7.7%	10	27.8%
KDR	4	16	17.8%	3	12.0%	4	25.0%	1	7.7%	8	22.2%
TPTE	21	16	17.8%	3	12.0%	3	18.8%	2	15.4%	8	22.2%
ZNF749	19	16	17.8%	4	16.0%	6	37.5%	2	15.4%	4	11.1%
BMP8B	1	15	16.7%	4	16.0%	2	12.5%	1	7.7%	8	22.2%
GLB1L	2	15	16.7%	6	24.0%	3	18.8%	1	7.7%	5	13.9%
SEMA5A	5	15	16.7%	2	8.0%	3	18.8%	3	23.1%	7	19.4%
CNTN4	3	14	15.6%	4	16.0%	4	25.0%	2	15.4%	4	11.1%
ZNF407	18	14	15.6%	1	4.0%	3	18.8%	2	15.4%	8	22.2%
LNX1	4	13	14.4%	3	12.0%	1	6.3%	1	7.7%	8	22.2%
PNLIPRP2	10	13	14.4%	3	12.0%	3	18.8%	3	23.1%	4	11.1%
MX2	21	12	13.3%	2	8.0%	2	12.5%	1	7.7%	7	19.4%
STK24	13	12	13.3%	2	8.0%	3	18.8%	2	15.4%	5	13.9%
AGBL5	2	11	12.2%	3	12.0%	3	18.8%	0	0.0%	5	13.9%

FBXO25	8	11	12.2%	0	0.0%	2	12.5%	2	15.4%	7	19.4%
MAPK7	17	11	12.2%	0	0.0%	3	18.8%	1	7.7%	7	19.4%
TSHZ1	18	11	12.2%	1	4.0%	3	18.8%	2	15.4%	5	13.9%
ZBTB5	9	11	12.2%	0	0.0%	3	18.8%	2	15.4%	6	16.7%
ABHD13	13	10	11.1%	1	4.0%	4	25.0%	3	23.1%	2	5.6%
AHNAK2	14	10	11.1%	2	8.0%	3	18.8%	1	7.7%	4	11.1%
EDIL3	5	10	11.1%	0	0.0%	2	12.5%	1	7.7%	7	19.4%
ERCC5	13	10	11.1%	2	8.0%	3	18.8%	2	15.4%	3	8.3%
HSF2BP	21	10	11.1%	1	4.0%	1	6.3%	1	7.7%	7	19.4%
MYO5B	18	10	11.1%	0	0.0%	2	12.5%	3	23.1%	5	13.9%
TRIM36	5	10	11.1%	0	0.0%	3	18.8%	2	15.4%	5	13.9%
VPS53	17	10	11.1%	0	0.0%	1	6.3%	2	15.4%	7	19.4%

Supplementary Table 2. Common copy number gain events in endometrioid ovarian carcinoma

	Chromosome	N	%	PR+/ER+		PR+/ER-		PR-/ER+		PR-/ER-	
				N	%	N	%	N	%	N	%
ZNF43	19	25	27.8%	5	20.0%	3	18.8%	3	23.1%	14	38.9%
AMY1C	1	19	21.1%	6	24.0%	3	18.8%	1	7.7%	9	25.0%
RABAC1	19	18	20.0%	2	8.0%	5	31.3%	1	7.7%	10	27.8%
GTF2H2C	5	16	17.8%	5	20.0%	3	18.8%	1	7.7%	7	19.4%
SEMA5A	5	16	17.8%	3	12.0%	1	6.3%	3	23.1%	9	25.0%
ZNF625-ZNF20	19	13	14.4%	2	8.0%	2	12.5%	1	7.7%	8	22.2%
STK24	13	12	13.3%	1	4.0%	1	6.3%	1	7.7%	9	25.0%
TMEM191B	22	12	13.3%	3	12.0%	2	12.5%	2	15.4%	5	13.9%
AHNAK2	14	11	12.2%	1	4.0%	1	6.3%	2	15.4%	7	19.4%
CCDC191	3	10	11.1%	1	4.0%	0	0.0%	2	15.4%	7	19.4%
CDK20	9	10	11.1%	5	20.0%	3	18.8%	1	7.7%	1	2.8%

Supplementary Table 3. Overlay of genomic subtypes and hormone receptor expression-based subtypes in endometrioid ovarian carcinoma

	PR-/ER-<br>N=36	PR-/ER+<br>N=13	PR+/ER-<br>N=16	PR+/ER+<br>N=25
***TP53*m**	15 (41.7%)	4 (30.8%)	2 (12.5%)	1 (4.0%)
***TP53*wt/*CTNNB1*wt**	15 (41.7%)	6 (46.2%)	5 (31.3%)	3 (12.0%)
***TP53*wt/*CTNNB1*m**	6 (16.7%)	3 (23.1%)	9 (56.3%)	21 (84.0%)

PR, progesterone receptor; ER, estrogen receptor; PR-high, PR+/ER+ or PR+/ER-; PR-low, PR-/ER+ or PR-/ER-; m, mutant; wt, wild-type

Supplementary Table 4. Frequency of *PIK3CA*, *ARID1A*, *PTEN* and *KRAS* mutation across hormone receptor-based endometrioid ovarian carcinoma subtypes

	PR-/ER-<br>N=36	PR-/ER+<br>N=13	PR+/ER-<br>N=16	PR+/ER+<br>N=25	P-value[a]	P-adj.
***PIK3CA*m (N=45)**	16 (44.4%)	3 (23.1%)	10 (62.5%)	16 (64.0%)	0.0343	0.1372
***PIK3CA*wt (N=45)**	20 (55.6%)	10 (76.9%)	6 (37.5%)	9 (36.0%)		
***ARID1A*m (N=31)**	11 (30.6%)	5 (38.5%)	5 (31.3%)	10 (40.0%)	0.8664	1
***ARID1A*wt (N=59)**	25 (69.4%)	8 (61.5%)	11 (68.8%)	15 (60.0%)		
***PTEN*m (N=21)**	5 (13.9%)	3 (23.1%)	6 (37.5%)	7 (28.0%)	0.1421	0.5684
***PTEN*wt (N=69)**	31 (86.1%)	10 (76.9%)	10 (62.5%)	18 (72.0%)		
***KRAS*m (N=25)**	11 (30.6%)	3 (23.1%)	4 (25.0%)	7 (28.0%)	1	1
***KRAS*wt (N=65)**	25 (69.4%)	10 (76.9%)	12 (75.0%)	18 (72.0%)		

[a]PR-high (PR+/ER+, PR+/ER-) vs PR-low (PR-/ER+, PR-/ER-) cases. Power to detect large (50% vs 10% mutation rate) and moderate (50% vs 20%) differences was 0.99 and 0.86 in a cohort of 90 patients with even case distribution, prior to adjustment for multiplicity of testing. Power to detect modest differences (50% vs 30%) was limited (approximately 0.49). P-adj, Bonferroni-adjusted P-value; m, mutant; wt, wild-type

Supplementary Table 5. Multivariable disease-specific survival analysis

		HR	95% CI	P-value
**PR status**	PR-high (PR+/ER+ or PR+/ER-)	0.16	0.04-0.71	0.016
	PR-low (PR-/ER+ or PR-/ER-)	reference	reference	reference
**Genomic subtype**	*TP53*m	reference	reference	reference
	*TP53*wt/*CTNNB1*wt	1.51	0.39-5.85	0.553
	*TP53*wt/*CTNNB1*m	0.75	0.17-3.27	0.703
**FIGO stage at diagnosis**	I	reference	reference	reference
	II	1.53	0.42-5.59	0.521
	III/IV	22.27	4.54-109.21	0.0001
**FIGO grade**	Grade 1	reference	reference	reference
	Grade 2	0.97	0.25-3.69	0.963
	Grade 3	0.97	0.34-2.78	0.953
**Residual disease**	Zero	0.34	0.11-1.01	0.052
	Macroscopic	reference	reference	reference
**Age at diagnosis**	Years	0.99	0.95-1.02	0.468

HR, hazard ratio; 95% CI, 95% confidence interval; PR, progesterone receptor; ER, estrogen receptor; PR-high, PR+/ER+ or PR+/ER-; PR-low, PR-/ER+ or PR-/ER-; m, mutant; wt, wild-type; FIGO, International Federation of Gynecology and Obstetrics.

Supplementary Table 6. Multivariable progression-free survival analysis

		HR	95% CI	P-value
**PR status**	PR-high (PR+/ER+ or PR+/ER-)	0.29	0.09-0.99	0.049
	PR-low (PR-/ER+ or PR-/ER-)	reference	reference	reference
**Genomic subtype**	*TP53*m	reference	reference	reference
	*TP53*wt/*CTNNB1*wt	1.27	0.37-4.38	0.708
	*TP53*wt/*CTNNB1*m	0.47	0.12-1.87	0.283
**FIGO stage at diagnosis**	I	reference	reference	reference
	II	2.37	0.74-7.57	0.144
	III/IV	33.91	7.26-158.48	<0.0001
**FIGO grade**	Grade 1	reference	reference	reference
	Grade 2	1.13	0.32-3.99	0.855
	Grade 3	0.77	0.27-2.19	0.626
**Residual disease**	Zero	0.49	0.19-1.31	0.156
	Macroscopic	reference	reference	reference
**Age at diagnosis**	Years	0.99	0.95-1.02	0.409

HR, hazard ratio; 95% CI, 95% confidence interval; PR, progesterone receptor; ER, estrogen receptor; PR-high, PR+/ER+ or PR+/ER-; PR-low, PR-/ER+ or PR-/ER-; m, mutant; wt, wild-type; FIGO, International Federation of Gynecology and Obstetrics

Supplementary Table 7. Clinicopathological characteristics of endometrioid ovarian carcinoma subtypes defined by combined PR and *TP53* mutation status

		**PR-high**	**PR-low/*TP53*wt**	**PR-low/*TP53*m**	**PR-low/*TP53*wt vs PR-low/*TP53*m**
**FIGO Stage at diagnosis**	I	19 (48.7%)	14 (46.7%)	4 (21.1%)	P=0.011
	II	17 (43.6%)	12 (40.0%)	5 (26.3%)	
	III/IV	3 (7.7%)	4 (13.3%)	10 (52.6%)	
	NA	2	0	0	
**Residual disease**	Zero	35 (92.1%)	24 (85.7%)	9 (50.0%)	P=0.022
	Macroscopic	3 (7.9%)	4 (14.3%)	9 (50.0%)	
	NA	3	2	1	
**Age at diagnosis**	Median years	56.0	61.5	62.0	P>0.2
**MATH score**	Median	29.7	32.7	54.7	P<0.001
**CNA count**	Median	11	24	98	P=0.001
**TMB**	Median	76	79	74	P>0.2

PR, progesterone receptor; ER, estrogen receptor; PR-high, PR+/ER+ or PR+/ER-; PR-low, PR-/ER+ or PR-/ER-; m, mutant; wt, wild-type; NA, not available; FIGO, International Federation of Gynecology and Obstetrics; MATH, mutant-allele tumor heterogeneity; CNA, copy number alteration; TMB, tumor mutational burden.

Supplementary Table 8. Recapitulation of integrated subtypes using immunohistochemistry for PR and p53

	**Integrated EnOC subtype**		
	PR-high	PR-low/*TP53*wt	PR-low/*TP53*m
**IHC classification** PR≥150	41	0	0
PR<150/ p53 wild-type	0	28	4
PR<150/ p53-aberrant	0	1	14

PR, progesterone receptor; PR≥150, PR histoscore ≥150; PR<150/p53 wild-type, PR histoscore <150 and wild-type p53 expression pattern; PR<150/p53-aberrant, PR histoscore <150 with aberrant p53 expression pattern
